# Urinary creatinine varies with microenvironment and sex in hibernating Greater Horseshoe bats (*Rhinolophus ferrumequinum*) in Korea

**DOI:** 10.1186/s12862-021-01802-z

**Published:** 2021-05-04

**Authors:** Heungjin Ryu, Kodzue Kinoshita, Sungbae Joo, Sun-Sook Kim

**Affiliations:** 1 School of Life Sciences, Ulsan National Institute of Science and Technology, UNIST- gil 50, 44919 Eonyang-eup, Ulju, Ulsan, Republic of Korea; 2 National Institute of Ecology, Geumgang-ro 1210, Maseo-myeon, 33657 Seocheon, Chungnam Republic of Korea; 3 Primate Research Institute, Kyoto University, 41-2 Kanrin, 484-8506 Inuyama, Aichi Japan; 4 Wildlife Research Center, Kyoto University, 2-24 Tanaka-Sekiden, 606-8203 Sakyo, Kyoto Japan

**Keywords:** Greater horseshoe bats, South Korea, Creatinine, Hibernation, Water stress

## Abstract

**Background:**

In temperate regions many small mammals including bats hibernate during winter. During hibernation these small mammals occasionally wake up (arouse) to restore electrolyte and water balance. However, field data on water stress and concentration of bodily fluids during hibernation is scarce. Urinary creatinine concentration has long been used to calibrate urinary hormone concentration due to its close correlation with urine concentration. Therefore, by investigating urinary creatinine concentration, we can estimate bodily fluid concentration. In this study, we investigated changes in urinary creatinine from greater horseshoe bats (*Rhinolophus ferrumequinum*) hibernating in abandoned mineshafts in two regions in South Korea.

**Results:**

We collected 74 urine samples from hibernating greater horseshoe bats from 2018 to 2019. We found that urinary creatinine concentration was higher in February and March and then declined in April. There were also indications of a sex difference in the pattern of change in creatinine concentration over the three months. Bats in the warmer and less humid mineshaft had higher urinary creatinine concentrations than bats in the colder and more humid mineshaft.

**Conclusions:**

These results indicate that hibernating bats face water stress as urinary concentration increases during winter and that water stress may vary depending on the microenvironment. Sex differences in behaviour during hibernation may influence arousal frequency and result in sex differences in changes in urinary creatinine concentration as hibernation progresses. Although further behavioural and endocrinal investigations are needed, our study suggests that urinary creatinine concentration can be used as a proxy to estimate the hydration status of bats and the effect of sex and environmental factors on arousal patterns during hibernation.

**Supplementary Information:**

The online version contains supplementary material available at 10.1186/s12862-021-01802-z.

## Background

Many small mammals in the temperate zone hibernate during winter [1], which enables them to overcome the energetic bottleneck caused by scarcity of food items and freezing temperatures. However, hibernation is also challenging for small mammals in that they have to rely mostly on stored body fat or cached food to survive the winter [2]. In addition to the limited availability of nutrients, small mammals must manage the water balance of their body fluids to survive through hibernation [3, 4]. As metabolic water alone is not sufficient for rehydration for small mammals, including bats, they adopt various strategies to maintain proper hydration status. For example, thirteen-lined ground squirrels (*Ictidomys tridecemlineatus*) reduce serum osmolality and suppress thirst, which helps them to maintain body fluid water balance during several months of hibernation [5]. Some bat species may form large clusters during hibernation to reduce evaporative water loss, and may also arouse to drink water inside or outside the hibernaculum [6–8]. Some bat species also feed on insects during winter, which will increase metabolic water formation and aid rehydration [9–12].

Small mammals also adopt various strategies to reduce evaporative water loss and energy expenditure [13, 14]. Among them, bats can provide a unique perspectives and information to the field of hibernation research [15, 16]. Their ability to fly allows bats to move between hibernacula, and can more freely select one with low ambient temperature (TA) and high relative humidity (RH) [17, 18], although such hibernacula are likely to be limited [19, 20]. Microclimatic constraints of a hibernaculum can be also counterbalanced by behavioural strategies, such as drinking and huddling so that sub-optimum hibernacula can be used for hibernation [7, 8]. As a result, the same bat species in different regions may differ in activity patterns and physiological parameters during hibernation depending upon the microclimate of the hibernaculum and the climate outside. Hibernating bats may also differ in activity patterns in relation to their sex and body condition (amount of fat storage). For example, male bats with more fat storage may engage in more mating activities during hibernation, which will cause shorter torpor bouts for those males [21]. Females may adjust torpor and arousal duration to save energy for foetal development after hibernation [22]. These differences in reproductive strategies between sexes may also result in individual variations in physiological parameters of hibernating bats.

Urine analysis has been used to estimate physiological conditions non-invasively, e.g., renal function and hormonal changes in primates [23]. Urinary creatinine is a by-product of metabolism and excreted through urine consistently [24]. Its concentration correlates with urine concentration, thus it has long been used to calibrate concentration of hormones and other components in urine [25]. High correlation between urinary creatinine and specific gravities has also been confirmed in flying foxes [26]. It is also known that urine concentration is correlated with blood concentration, probably reflecting the hydration status of bats [27]. Therefore, by investigating the urinary creatinine concentration, we can estimate urine concentration, and hydration status of hibernating bats [28]. Creatinine measurement is also cost-effective and requires only a small volume of urine (less than 10ul), which is crucial in the case of small insectivorous bats, as they expel only a small volume of urine (less than 40ul [28]). However, despite such advantages, creatinine measurement has rarely been applied in studies of bats, apart from flying foxes.

In this study, we analysed urinary creatinine in hibernating greater horseshoe bats (*Rhinolophus ferrumequinum*) in South Korea to investigate urine concentration as an indicator of water stress during hibernation. We hypothesized that urinary creatinine concentration may vary in relation to hibernaculum environment and sex. If individual bats become dehydrated during torpor, they need to intake water by drinking or by renal water reabsorption. As drinking requires body movement, including flight, which increases energy expenditure, renal water reabsorption restored during arousal might be an adequate strategy for rehydration during hibernation. Renal water reabsorption will result in higher concentration of urine and urinary creatinine. We therefore predict that urinary creatinine concentration will be higher during winter than spring. In addition, water vapor pressure and other abiotic factors, such as TA and RH of the hibernaculum may result in different degree of water stress for hibernating bats. To compensate for environmentally induced water stress, hibernating greater horseshoe bats may adjust arousal frequency and/or duration. Therefore, bats in a hibernaculum with a higher TA and lower RH may have higher urinary creatinine concentration. In addition, if males and females behave differently to improve their reproductive success [22] it could result in sex differences in urine concentration because of differences in metabolic water production.

Greater horseshoe bats arouse once a week on average during hibernation in the UK [29]. This arousal may be related to the need for water or energy intake [29], movement between hibernacula [30], or mating behaviours [31, 32]. However, the reasons for arousal during hibernation have not yet been fully elucidated from a physiological perspective. This study presents a preliminary investigation into whether dehydration faced by hibernating bats can be traced through urinary creatinine concentrations. We predicted that greater horseshoe bats would face dehydration that causes higher urinary creatinine concentration during hibernation, as in other hibernating bats [3, 7]. We also predicted that the creatinine concentration would decrease in early spring because of decreasing torpor bout lengths alongside increases in ambient temperature [29]. If microenvironment or sex differences caused difference in energy expenditure or drinking frequency, creatinine concentration might vary between different hibernacula or between sexes.

## Results

**Fig. 1:**
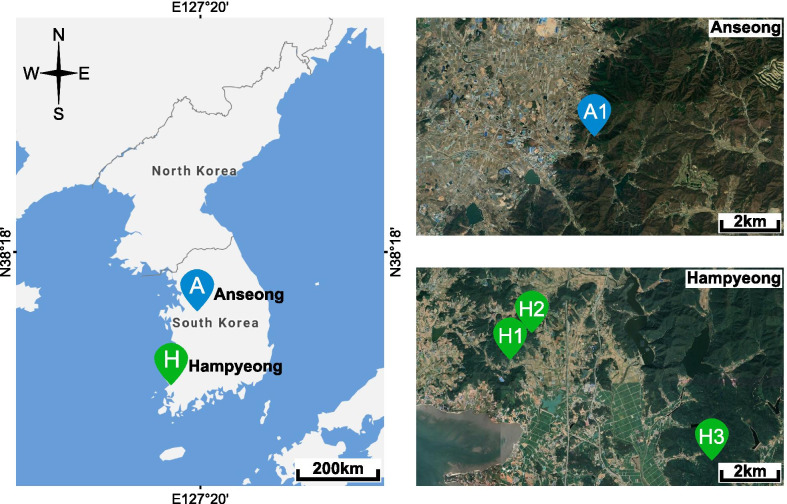
Sampling locations in South Korea. The distance between mineshaft A1 (Anseong) and mineshaft H1 (Hampyeong) is around 210 km. Maps were extracted from google maps

**Fig. 2:**
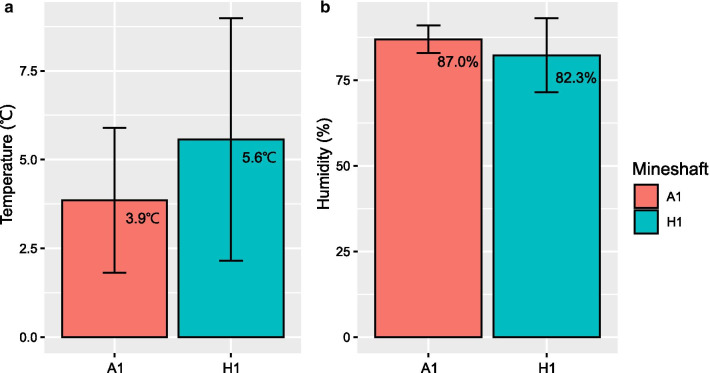
Mean daily TA and mean daily RH in the two mineshafts, A1 and H1. Error bars are standard deviations

To investigate the effect of microenvironment variations of mineshafts, we compared daily mean TA and RH (measured hourly and averaged daily) in the two mineshafts in Anseong (A1) and Hampyeong (H1), (Fig. 1). Bats in A1 used hibernation locations between around 50 and 150 m in from the entrance. However, in H1 most bats hibernated at the point furthest from the entrance (ca. 30 m). Since TA and RH data were only available for January to April 18 2019, we could not compared TA and RH for 2018. The mean TA from January 1 to April 18, 2019 was 3.9 ± 2.0℃ (0.6 to 8.1℃) in A1 and 5.6 ± 3.4℃ (-3.3 to 10.5℃) in H1 (Fig. 2). The mean RH was 87.0 ± 4.0 % (78.9 to 94.5 %) for A1 and 82.3 ± 10.9 % (55.2 to 97.0 %) for H1 (Fig. 2). TA was higher in H1 than A1 (paired t-test; t = 9.00, p < 0.001), and RH was higher in A1 than H1 (paired t-test; t = 6.40, p < 0.001). A Levene’s test confirmed greater variance of TA (F_1,214_ = 18.48, p < 0.001) and RH (F_1,214_ = 60.51, p < 0.001) in H1 than A1 (Additional file 1: Figure S1).

**Fig. 3:**
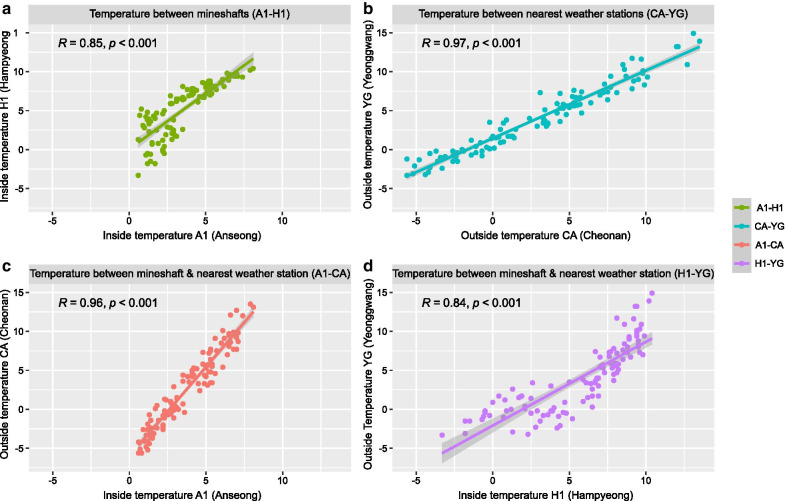
Relationship between TA and correlation coefficients: **a** between the daily TA of two mineshafts, A1 and H1; **b** between the nearest weather stations, CA and YG; **c** between A1 and CA; **d** between H1 and YG

**Fig. 4:**
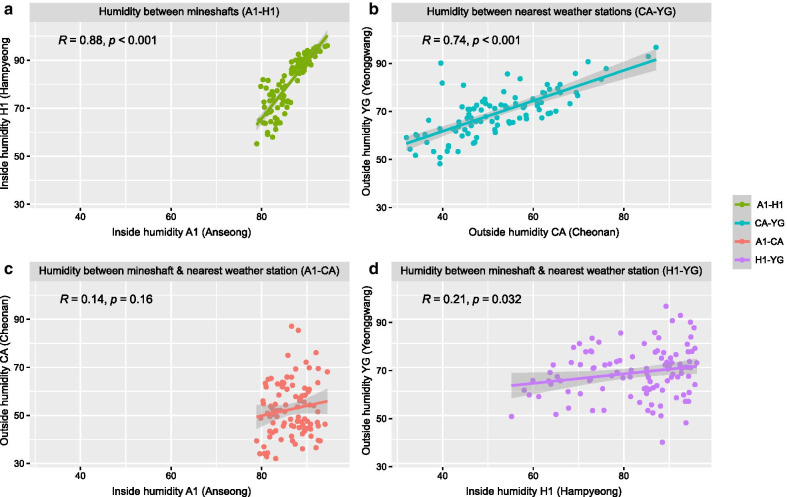
Relationship between RH and correlation coefficients: **a** between the daily RH of two mineshafts, A1 and H1; **b** between the nearest weather stations, CA and YG; **c** between A1 and CA; **d** between H1 and YG

The TA in the two mineshafts was strongly correlated (Fig. 3a), even though they are over 210 km apart. The TA was also strongly correlated at each of the weather stations nearest to the mineshafts (CA and YG, Fig. 3b). In addition, the TA at each of the mineshafts was strongly correlated with the TA at the nearest weather stations (Fig. 3c and d). These results indicate that the TA inside the two mineshafts was influenced by the outside temperature. The RH was also highly correlated between the two mineshafts, and between the two weather stations (Fig. 4a and b). However, the RH of mineshaft A1 was not correlated with the RH at the nearest weather station CA, although the RH of H1 was weakly correlated with the RH at the nearest weather station YG (Fig. 4c and d). In contrast to the TA inside the mineshafts, RH was not correlated with the outside RH. These results indicate that the RH inside the mineshaft was more independent from the air flow from outside than the TA inside the mineshaft.

**Fig. 5:**
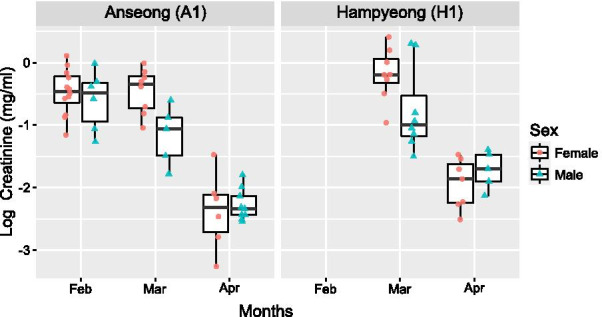
Creatinine concentrations (natural log-transformed) between mineshafts A1 (left) and H1 (right). Lines in boxes: the median, the top and bottom of boxes: the first and third quartiles, vertical lines (whiskers): the maximum (or the minimum) value within 1.5 times interquartile range above 75th (or below 25th) percentiles, dots: natural log-transformed creatinine concentration of each urine from females (circle) and males (triangle)

In total, we collected 74 urine samples from 33 males and 41 females (Table 1). The mean urinary creatinine concentrations (± SD) during the main hibernation (Feb to Mar) and early active period (Apr) were 0.65 ± 0.32 mg/ml (N = 47) and 0.13 ± 0.06 mg/ml (N = 27) respectively. To investigate changes in urinary creatinine concentration of bats in the two mineshafts, we ran a generalized linear mixed model (GLMM – see methods for further details). This showed that creatinine concentrations from the urine collected in H1 were higher than those collected in A1 (Fig. 5; Table 2). There was no sex difference in creatinine concentration. Urinary creatinine concentration was lower in April than in February and March. Although, there was an indication of a sex difference patterns of change (interaction term in the model) in urinary creatinine between February to March, the interaction term did not reach statistical significance (Fig. 5; Table 2).

**Table 1:** The number of urine samples and sampling locations

Date	Periods	Male	Female	Location	Hibernaculum
2018-02-22	Hibernation	4	8	Anseong	A1
2018-03-13	Hibernation	5	8	Anseong	A1
2018-04-17	Active	5	7	Hampyeong	H1
2018-04-18	Active	9	6	Anseong	A1
2019-02-22	Hibernation	2	4	Anseong	A1
2019-03-11	Hibernation	8	8	Hampyeong	H2, H3
Sum		33	41		

The number of urine samples from males and females are in the table. February and March are main hibernation periods (late winter) and April is the early active period (spring)

**Table 2:** The GLMM summary results that investigated the effect of sex, month, and the two mineshafts on urinary creatinine concentration (natural log-transformed)

	Est	SE	t	P	95 % CI (lower, upper)
(Intercept)	− 0.47	0.13	− 3.72	< 0.001*	− 0.72, − 0.20
Sex (M)	− 0.13	0.22	− 0.61	0.544	− 0.58, 0.29
Month (Mar)	− 0.06	0.18	− 0.31	0.757	− 0.42, 0.31
Month (Apr)	− 1.89	0.19	− 10.14	< 0.001*	− 2.28, − 1.52
Mineshaft (H1)	0.41	0.12	3.42	0.001*	0.15, 0.66
Sex (M): Month (Mar)^a^	− 0.51	0.27	− 1.86	0.067^b^	− 1.03, − 0.03
Sex (M): Month (Apr)^a^	0.27	0.28	0.98	0.333	− 0.24, 0.81

In the model sample collection years (2018 and 2019) were included as a random variable

^a^Interaction term between sex and month

^b^Not significant but indicates possible sex-dependent changes in urinary creatinine over months

*: Statistically significant (p < 0.05)

## Discussion

Our study is the first to report changes in urinary creatinine in wild bats in hibernacula from late winter to early spring. As expected, creatinine concentration (urine concentration) was higher during the main hibernation (Feb to Mar) than in the early active period (Apr). The higher concentration of urinary creatinine during hibernation (5-times) than spring implies that hibernating greater horseshoe bats face dehydration (water stress) that may increase renal water reabsorption. By spending longer periods in torpor, hibernating bats can reduce the energy consumption required for arousal. But as the period in torpor gets longer, cumulative evaporative water-loss will increase the concentration of bodily fluids [3]. To compensate for water loss during longer torpor periods, hibernating bats can increase renal water reabsorption during arousal, as found in marmots and ground squirrels [13, 33]. This will then result in higher urinary creatinine in torpid bats during hibernation. Therefore, higher urinary creatinine concentration in hibernating bats in the current study may reflect high body fluid concentration from evaporative water loss that might be correlated with torpor duration. In contrast, lower creatinine in April, may indicate shorter torpor durations in April that are related to feeding activities that increase with the outside temperature [29].

Previous studies on *Myotis velifer* demonstrated that these bats do not produce hypertonic urine even during hibernation [28, 34]. This is different from our findings for greater horseshoe bats. If hibernating bats accumulate serum creatinine from metabolism (thermogenesis) during torpor, as in ground squirrels [35] and dormice [36], the accumulated serum creatinine will be secreted to urine during arousal when glomerular filtration rate becomes normal [13]. In such cases urinary creatinine concentration in the bladder will be high, but may not be correlated with the urine concentration (osmolality). If this is the case for greater horseshoe bats, torpor bout length and metabolism during torpor will be more important determining factors for higher urinary creatinine concentration than the evaporative water loss that causes dehydration. Further investigations on serum and urinary osmolality and creatinine concentration are necessary to clarify the relation between urinary concentration and urinary creatinine concentration during hibernation.

The creatinine difference between A1 and H1 may reflect differences in the microenvironment between the two mineshafts. Although data were only available for 2019, the mean and variance of daily TA of H1 were greater than A1 (Fig. 2). In contrast, the mean daily RH was lower and the variance was greater in H1 than A1. Given that these microenvironment parameters were consistent with a previous study conducted in the same place [30], the higher creatinine concentrations in H1 might be related with higher TA and lower RH in H1 that could cause higher evaporative water loss [37]. However, we could not rule out the possibility that the higher creatinine concentrations in H1 were related to frequent or longer arousal that can result in increase of glomerular filtration rate and an increase in the accumulation of urinary creatinine in bladder [13]. In addition, as hibernating greater horseshoe bats can shift their roosting location within and between hibernacula [30, 38], the urinary creatinine differences between A1 and H1 might not result solely from microenvironment differences. Further investigation on arousal frequency and metabolic rate between the two mineshafts will elucidate the reason for the creatinine differences between the two hibernacula.

Our results suggest that the creatinine concentration decrease may start earlier in males than females, with the interaction between sex and month in the GLMM approaching significance. The tendency was similar across the year as seen in Additional file 1: Figure S3. A sex difference in urinary creatinine changes over months may be related to activity differences at the end of the hibernation period (Mar) [31, 32]. Direct and indirect evidence suggests that mating during hibernation is common in small insectivorous bats, including greater horseshoe bats [31, 39, 40]. Males can even mate with torpid females [41]. Ovulation in female greater horseshoe bats in Korea probably occurs in April [42, 43]. Therefore, males may get extra benefit if they can mate with pre-ovulating females in March. Although we did not witness spring mating, if males could mate before the females ovulate in spring, they could increase their chances of siring offspring.

Although Park et al. in 2000 [29] reported no sex difference in winter activities of greater horseshoe bats in the UK, there were indications of monthly variations in activity patterns between sexes even in their study (see Fig. 1 of their study [29]). Therefore, lower creatinine concentration for males in March in the current study may indicate more frequent or longer arousal duration of males that entails an increase in body fat metabolism, and consequent increase in metabolic water generation. In addition, males may alter their arousal frequency or duration of arousal to save energy, or to increase energy intake, depending on their body condition [29]. For example, males with less fat storage at the end of hibernation periods need to arouse to find prey items, or remain in torpor alone in a colder area to decrease metabolic rate. Further behavioural and physiological monitoring and hormonal investigations are needed to examine these possibilities.

Differences in muscle mass were unlikely to have been the cause of the sex difference in creatinine changes over the months in this study. Although females were heavier than males during late winter (Feb to Mar) in our study population [Kim, unpublished data], as has been reported in a UK population [38], no sex-specific patterns in changes of body weight in February and March were found [Kim, unpublished data]. Also, as muscle mass decreases during hibernation in bats [44], it may even counter the increase of creatinine. Therefore, it is unlikely that the earlier decrease of creatinine among males resulted from a sex difference in body muscle mass. However, to clarify this issue there needs to be further investigation into the relationship between urinary creatinine concentration and body weight during hibernation. This will also allow us to understand better the relationship between body condition and energy expenditure that may reflect a trade-off between energy saving by decreasing basal metabolism rate (BMR) and metabolic water supply by increasing BMR [22, 45].

## Conclusions

The current study presents a novel methodological technique for the quantification of physiological challenges, e.g., water stress, energy expenditure and metabolic wastes, during hibernation in bats. Although the sample size was small, this study has demonstrated the feasibility of urinary monitoring as a method to trace hydration status (water-stress). This study also provided evidence of higher urinary creatinine concentration that might reflect different water stress depending on hibernaculum environment in small insectivorous bats. Although not statistically significant, the sex difference in creatinine changes between months implies a behavioural or energy expenditure difference between sexes during hibernation. Further behavioural and endocrinological investigations will clarify the effect of the sex and behaviours on urinary creatinine concentration.

## Methods

### Study sites and subjects

We collected urine samples from greater horseshoe bats in two regions of South Korea in 2018 and 2019 (Fig. 1). Mineshaft A1 in Anseong is about 350 m long and running water is available throughout the winter. Mineshaft H1 in Hampyeong is only about 35 m long and has no running water inside from January to February [30]. We logged the ambient temperature (TA) and relative humidity (RH) in A1 and H1 every hour from January 1 to April 18, 2019 using EL-USB-2-LCD + dataloggers (Lascar Electronics, UK). We were not able to collect TA and RH in 2018. We calculated daily mean TA and RH, by averaging TA and RH of every hour of the day. The microenvironments of the three mineshafts in Hampyeong (H1, H2 and H3) are similar during winter and bats are known to switch between them [30]. February and March were categorized as the main hibernation period (late winter) and April as the early active period (spring), as mean daily temperature reached 10 ℃ in April in the study area (Additional file 1: Figure S3) and the number of moths and their activity increase rapidly in April in South Korea [46, 47].

### Urine sampling and analysis

We conducted urine sampling just once a month to minimize disturbance to the bats. The numbers of urine samples were inconsistent between months and years because of fluctuating numbers of bats in the mineshafts. Most bats in A1 were out of hand’s reach in 2019, so we could only collect six samples in February, and none in March. We tried additional sampling in H1 in March 2019 to compensate for no sampling from A1. However, this was also not successful due to the small number of bats present, so we collected additional urine samples from H2 and H3. We could not collect urine in either of the two regions in April 2019 because most of the bats were roused by our presence.

To collect urine, one researcher took a hibernating or torpid bat gently in a gloved hand and held it for up to a few minutes until they urinated. If it had not urinated after 5 min, we put it back in the place it had been taken from. We banded sampled bats with a 35mm metal ring on the forearm, but this was done only in February and April 2018 (online repository data [48]) to keep the time we spent in the hibernacula to a minimum. When they urinated, we collected their urine directly from their genitalia using a 1.5ml micro-tube. We temporarily stored urine samples in an icebox at the field sites then stored them in a -80℃ deep-freezer until analysis. The maximum storage period in the deep-freezer before urinary analysis was 7 months. We used a creatinine kit (Abcam ab204537; Jaffe reaction based [49]) to measure urinary creatinine concentration. As the amount of urine was small (usually less than 25 µl), we diluted samples 20 times with deionized water. One particularly small sample was diluted 29.5 times. Before dilution, all samples were centrifuged 6000 rpm (3381rcf) for 1 min to remove any suspended matter. The intra-assay coefficient of variation (CV) of the creatinine analysis was 5.30 % (online repository data [48]).

### Statistical analysis

We investigated microenvironment variations between A1 and H1, by comparing daily mean and variance of TA and RH using a paired t-test and Levene’s test respectively. To investigate the effect of microenvironment on urine concentration we compared creatinine concentration between mineshafts and sexes over months by building a generalized linear mixed model (GLMM). In the model, urinary creatinine concentration (natural log-transformed) was the response variable. Sex (males and females), month (Feb, Mar, and Apr) and location of mineshafts (A1 and H1) were the explanatory variables in the model. Year of urine collected (2018 and 2019) was included as a random variable in the model given the strong correlations between the inside temperature and outside temperature (Figs. 3 and 4). We used the “summary” function in R to further investigate the model coefficients. To calculate confidence interval, we used the “confint” function in R. Samples were considered independent as individuals were very unlikely to have been sampled twice. This is because there were hundreds of bats were present in A1 (the only site that was sampled more than once) and all bats sampled in February and April were banded (online repository data [48]).

All statistical tests and graphics were done in R 4.0.2, using “lme4 [50]”, “lmerTest [51]”, “car [52]”, “ggplot2 [53]” packages. All assumptions of the GLMM were examined by using statistical tests (Shapiro-Wilk) and visual inspections (Q-Q plot) of the error (residuals) distribution. There was no violation of the assumptions. The maximum value of generalized variance inflation factor (GVIF) was 2.14 which indicates no serious multicollinearity between independent variables.

## Supplementary Information

**Additional file 1: Figure S1.** Daily ambient temperature, TA (a) and relative humidity, RH (b) of A1 and H1 in 2019. **Figure S2.** Ambient temperature (top) and relative humidity (bottom) at the nearest weather stations in Cheonan (CA) and Yeonggwang (YG) in 2018 and 2019. The nearest weather station to A1 (Anseong) is 17.6km away in Cheonan (N36°45’45.576” E127°17’33.071), and for H1 (Hampyeong), it is 18.2km away in Yeonggwang (N35°17’1.932” E126°28’39.071”). Temperature data were collected by the automatic weather system (AWS) managed by the Korean Government (https://data.kma.go.kr/). **Figure S3.** Creatinine concentrations in 2018 and 2019 (N = 74). Lines in boxes: the median, the top and bottom of boxes: the first and third quartiles, dots: creatinine concentration of each urine from females (circle) and males (triangle).

## Data Availability

The datasets generated and/or analysed during the current study are available at Figshare online repository [48]: https://figshare.com/s/3c0301e8fb689807b76d.

## Abbreviations

TA: Ambient temperature
RH: Relative humidity
A1: Mineshaft 1 in Anseong
H1, H2, H3: Mineshaft 1, 2, 3 in Hampyeong
UK: United Kingdom
GLMM: Generalized Linear Mixed Model
BMR: Basal metabolism rate
