# Supplementary material for: Urinary creatinine varies with microenvironment and sex in hibernating Greater Horseshoe bats (Rhinolophus ferrumequinum) in Korea
Source: BMC Ecol Evol. 2021 May 4;21:77. doi: 10.1186/s12862-021-01802-z (PMC8094569; doi:10.1186/s12862-021-01802-z)
Supplement: Supplementary file 1 — Additional file 1: Figure S1. Daily ambient temperature, TA (a) and relative humidity, RH (b) of A1 and H1 in 2019. Figure S2. Ambient temperature (top) and relative humidity (bottom) at the nearest weather stations in Cheonan (CA) and Yeonggwang (YG) in 2018 and 2019. The nearest weather station to A1 (Anseong) is 17.6km away in Cheonan (N36°45’45.576” E127°17’33.071), and for H1 (Hampyeong), it is 18.2km away in Yeonggwang (N35°17’1.932” E126°28’39.071”). Temperature data were collected by the automatic weather system (AWS) managed by the Korean Government (https://data.kma.go.kr/). Figure S3. Creatinine concentrations in 2018 and 2019 (N = 74). Lines in boxes: the median, the top and bottom of boxes: the first and third quartiles, dots: creatinine concentration of each urine from females (circle) and males (triangle). [file 12862_2021_1802_MOESM1_ESM.docx]

# Electronic supplementary material

**Urinary creatinine varies with microenvironment and sex in hibernating Greater Horseshoe bats (Rhinolophus ferrumequinum) in Korea**

Heungjin Ryu^1, 2, 3^, Kodzue Kinoshita^4^, Sungbae Joo^2^, Sun-Sook Kim^2^*

1. School of Life Sciences, Ulsan National Institute of Science and Technology, UNIST-gil 50, Eonyang-eup, Ulju, Ulsan 44919, Republic of Korea
2. National Institute of Ecology, Geumgang-ro 1210, Maseo-myeon, Seocheon, Chungnam 33657, Republic of Korea
3. Primate Research Institute, Kyoto University, 41-2 Kanrin, Inuyama, Aichi 484-8506, Japan
4. Wildlife Research Center, Kyoto University, 2-24 Tanaka-Sekiden-cho, Sakyo, Kyoto 606-8203, Japan

Authors for correspondence:

Sun-Sook Kim

E-mail: [sskim@nie.re.kr](mailto:sskim@nie.re.kr)

## Figure S1.

Daily ambient temperature, TA (a) and relative humidity, RH (b) of A1 and H1 in 2019.

## Figure S2.

Ambient temperature (top) and relative humidity (bottom) at the nearest weather stations in Cheonan (CA) and Yeonggwang (YG) in 2018 and 2019.

The nearest weather station to A1 (Anseong) is 17.6km away in Cheonan (N36°45'45.576" E127°17'33.071), and for H1 (Hampyeong), it is 18.2km away in Yeonggwang (N35°17'1.932" E126°28'39.071"). Temperature data were collected by the automatic weather system (AWS) managed by the Korean Government (<https://data.kma.go.kr/>).

## Figure S3.

Creatinine concentrations in 2018 and 2019 (N=74). Lines in boxes: the median, the top and bottom of boxes: the first and third quartiles, dots: creatinine concentration of each urine from females (circle) and males (triangle).
